# Safe Use of Flammable Endotracheal Tubes During Anesthesia for Laryngeal Laser Surgery-Report of 1024 Cases and a Brief Literature Review

**Published:** 2019-07

**Authors:** Masood Mohseni, Farzad Izadi

**Affiliations:** 1 ***Department of Anesthesiology, Iran University of Medical Sciences, Tehran, Iran.***; 2 ***Department of Otolaryngology, Iran University of Medical Sciences, Tehran, Iran.***

**Keywords:** Anesthesia, Airway management, Surgery, Endotracheal, Equipment safety, Laser therapy.

## Abstract

**Introduction:**

One of the major concerns in laryngeal laser surgery is the risk of airway fire. The introduction ofwrapped tubes and metal tubes has reduced the fire hazards. However, these tubes are expensive and do not provide convenient access to the surgical field. There are few laboratory studies addressing the resistance of polyvinylchloride tubes against ignition in the given circumstances. Nevertheless, its safety should be approved in clinical practices.

**Materials and Methods:**

This retrospective studyevaluated the airway management on 1024 patients undergoing laryngeal laser surgery. The data collection included the information about the type of endotracheal tube (ETT), mode of ventilation, and airway hazards (e.g., tube ignition).

**Results:**

Polyvinylchloride tubes and conventional positive pressure ventilation was applied for most of the patients (84.1%). The tube cuff was pierced with laser beam in 22 cases (2.5%). However, there was no case of ETT ignition or airway fire.

**Conclusion:**

Polyvinylchloride tubes can be safely used in this subset of surgeries pending meticulous attention to the safety recommendations.

## Introduction

Laser surgery of the larynx offers several advantages over conventional surgical methods, including a bloodless operative field and microscopic precision (1). However, the anesthetist faces special issues in the management of these patients. Some of these issues are the preoperative evaluation of the degree of airway obstruction, possible behavior of the laryngeal mass with respect to airway obstruction and bleeding tendency during the operation, cooperation with the surgeon in the shared field, airway fire protocol, reduction of the inhalational hazards due to plume and eye protection against direct traumatic effects of laser beam (2-4). One of major concerns in laryngeal laser surgery is the risk of airway fire. While airway fire is relatively uncommon, it is very serious and can result in severe morbidities and even mortality. The introduction of wrapped tubes, metal tubes, and jet ventilation techniques using a needle or metal tube have reduced the fire hazard; however, each method has its own set of problems. When endotracheal intubation is intended, its flammability is a great concern. There are few studies addressing the safety of polyvinylchloride (PVC) tubes for laser surgery. They are mainly designed in a mechanical laboratory model and have suggested conflicting results (5,6). 

Hereby, this study was conducted to review the recommendations on the anesthetic management of microlaryngeal laser surgery in light of our own experience to provide a feasible and simultaneously safe plan of anesthesia administration.

## Materials and Methods

This retrospective study was a report of 1024 cases of laryngeal laser surgery over a period of five years. The patients of either gender were within the age range of 2-91 years. The procedures with endotracheal tube (ETT) included a variety of pathologies, such as laryngeal web, benign mass lesions, phonosurgery for pitch alteration, and ablative surgery for laryngeal carcinoma. The application of laser**-**safe ETTs was for the educational purposes for residents and the type and size of laryngeal pathology did not influence the choice of ETTs. The most usual ETT for adults was PVC cuffed tube with ID of 5.5. The tube was kept in place during laser surgery and removed at the end of operation after the reversal of neuromuscular blockade.

The plan for initial airway management and intubation was based on the preoperative assessments, including the radiologic evaluations, stroboscopy, and clinical examinations (e.g., indirect laryngoscopy). In case difficult airway was suspected, the implementation of awake intubation with fiberoptic bronchoscope, videolaryngoscope or laryngoscopy with Macintosh blade with appropriate size was considered. Otherwise, patients were premedicated with midazolam and fentanyl, general anesthesia was induced with propofol and atracurium. For the maintenance of anesthesia, total intravenous anesthesia (TIVA) with propofol 2 mg.kg^-1^plus atracurium 0.05 mg.kg^-1^ was applied. None of the patients received volatile agents for the maintenance of anesthesia. The fraction of delivered oxygen (FiO_2_) was maintained below 40% using a mixture of oxygen and air. 

## Results

In 862 patients (84.1%), PVC endotracheal tubes (ETT) and conventional positive pressure ventilation were administered. In the remaining patients, tubeless jet ventilation (141 patients) or laser safe stainless steel ETT (21 patients) was applied. Of all, in 23 cases, including 22 PVC and 1 laser safe tube, the cuff was pierced with laser beam. However, there was no case of ETT ignition or airway fire (Table.1).

**Table 1:** Summary of the study findings

	**Types of airway management**		
	**Tubeless jet ventilation**	**PVC endotracheal tube**	**Laser safe endotracheal tube**
Number of patients	141 (13.7)	862 (84.1)	21 (2.1)
Pierced cuff	0 (0)	22 (2.5)	1 (4.7)
Airway ignition	0 (0)	0 (0)	0 (0)

Data are presented as frequency (%)

## Discussion

To conduct a safe anesthesia method for laryngeal laser surgery, the anesthetist should be familiar with the application of small-sized laser-safe tubes(5,7), tubeless techniques, jet ventilation(8,9), and apneic methods (10).

When endotracheal intubation is intended, the fuel for airway fire is present (The endotracheal tube becomes a ready source of fuel if ignited by a laser).Precautions to reduce the risk of ETT ignition include the distal placement of ETT cuff, maintenance of less than 40% oxygen concentration, application of wet gauze in the surgical field, filling the ETT cuff with water instead of air, and careful attention to the laser reflections. 

The application of powerful smoke suction improves the visibility of surgical field and reduces the risk of displaced laser irradiation. Other safety measures, such as water syringe for rescue treatment, should also be available. Total intravenous anesthesia is the recommended method for maintenance of anesthesia. To reduce the risk of airway fire, the applied power of carbon dioxide (CO_2_) laser was limited to 2-3 W for benign lesions preferably with super-pulse mode. In the case of ablative surgeries, specifically in cancer patients, 8W continuous mode laser was routinely used. If cartilage ablation was required (i.e., arytenoidectomy**)**, 10 W continuous mode usually would suffice. However, up to 15 W continuous mode was rarely utilized. The application of higher powers increased the risk of thermal injury and postoperative tissue fibrosis.In this study, the PVC tube size 5.5 was commonly applied for adult patients. This size made a straightforward surgical access without significant airway resistance complicating the mechanical ventilation. In the case of narrowed glottis opening due to mass effect and in children, smaller appropriate sizes were selected. Laser safe tubes, including wrapped tubes and metal tubes, are much more expensive than conventional PVC tubes. Moreover, metal tubes (e.g., stainless steel ETTs) have more external diameters with comparable lumen size, which impairs the exposure of laryngeal pathology. Their nearly rigid firmness makes them hardly relocatable and intraoperative surgical maneuvers difficult. 

## Conclusion

According to the obtained results of the current study, it can be concluded that PVC tubes can be safely used in laryngeal laser surgeries pending the wise cooperation of anesthetist and surgeon in the shared field and meticulous attention to the other safety recommendations (11,12). 

Further studies are warranted to examine the flammability of ordinary used tubes in the applied setting.
